# Understanding Cancer Health Disparities

**DOI:** 10.3390/cancers18030476

**Published:** 2026-01-31

**Authors:** Jun Zhang, Wei Du, Youping Deng, Herbert Yu, Peiwen Fei

**Affiliations:** 1 Department of Laboratory Medicine and Pathology, Mayo Clinic Foundation, Phoenix, AZ 55905, USA; zhang.jun@mayo.edu; 2 UPMC Hillman Cancer Center, University of Pittsburgh, Pittsburgh, PA 15232, USA; wed41@pitt.edu; 3 John A. Burns School of Medicine, University of Hawaii, Honolulu, HI 96813, USA; dengy@hawaii.edu; 4 The University of Hawaii Cancer Center, University of Hawaii, Honolulu, HI 96813, USA

**Keywords:** cancer health disparities, genomic stability, cancer susceptibility, DNA damage, gene mutations, epigenetic modifications, methylation, social determinants of health

## Abstract

Cancer health disparities represent profound inequalities in incidence, outcomes, and survivorship across populations. While traditionally examined through distinct lenses of either molecular biology or social epidemiology, these disparities arise from the complex interplay of genetic susceptibility, epigenetic dysregulation, and social determinants of health (SDoH). This review proposes that DNA damage and genomic instability serve as a critical mechanistic bridge, integrating exposures from the societal level to cellular dysfunction. We synthesize evidence demonstrating how SDoH—such as systemic inequities, environmental exposures, and chronic stress—converge with genetic and epigenetic factors to disproportionately increase DNA damage burden, impair repair mechanisms, and accelerate tumorigenesis in marginalized communities. Using the elevated gastrointestinal cancer rates among Native Hawaiians and Pacific Islanders (NH/PI) as a case study, we illustrate how historical, environmental, and socioeconomic factors interact with biological pathways to drive disparities. The review highlights key advances in DNA damage research—from somatic mutation theory to the modern understanding of chronic genomic stress—and explores how innovations in single-cell genomics, biomarker discovery, and computational modeling can unravel disparity etiologies. We argue that a translational framework linking social exposure data to molecular biomarkers of DNA damage is essential for moving beyond descriptive disparities to mechanistic understanding. Ultimately, addressing cancer equity requires interdisciplinary strategies that bridge molecular oncology, public health, and community-engaged research, targeting the root causes where social inequities become biologically embedded as genomic instability.

## Simple Summary

Cancer health disparities—differences in cancer measures including new cancer cases, all existing cases, mortality, survivorship, screening rates, stage at diagnosis, etc.—are influenced by complex interactions between biological, environmental, and social factors. DNA damage studies provide critical insights into these disparities by unraveling how genetic instability, repair deficiencies, and environmental exposures disproportionately affect marginalized groups. Below, we discuss the factors eventually driving genomic instability leading to cancer susceptibility, and how these factors are integrated in the real world and lead to cancer health disparities, such as among Native Hawaiians/Pacific islanders (NH/PI).

## 1. Introduction

Modern oncology recognizes that genomic instability—a state of increased DNA mutation rates and chromosomal aberrations—is a defining feature of cancer. It fuels tumor evolution, heterogeneity, and therapy resistance [1,2,3,4,5]. This instability is not solely a product of inherited or somatic mutations, but the final common pathway for a wide array of disruptions, from molecular to societal [6,7,8]. However, for decades, the somatic mutation theory has dominated. It posits that cancer arises from accumulating DNA mutations in key genes (e.g., oncogenes, tumor suppressors), leading to uncontrolled clonal expansion [9,10,11]. The modern synthesis recognizes that while mutations are nearly universal in cancer, the path to malignancy is paved by the chronic, systemic dysregulation caused by persistent DNA damage and the resulting tissue-level chaos [7,10,12,13]. The most productive approach is to see DNA damage as the molecular mediator through which social determinants, environmental exposures, and genetic predispositions converge to initiate cancer within a destabilized tissue system.

DNA damage, arising from endogenous processes such as oxidative metabolism or exogenous insults like ultraviolet radiation and chemical mutagens, poses a persistent threat to genomic integrity. Unrepaired or misrepaired damage can lead to mutations, chromosomal instability, and diseases ranging from cancer to neurodegeneration [14,15,16]. Over the past decade, advancements in genome-wide profiling, single-cell resolution technologies, and computational modeling have revolutionized our understanding of DNA damage detection, repair mechanisms, and their broader implications in health and disease [17,18]. These innovations now enable researchers to map damage at nucleotide precision, dissect repair pathway dynamics in heterogeneous cell populations, and uncover novel therapeutic vulnerabilities in cancer and beyond.

Recent studies highlight the dual role of DNA damage response (DDR) pathways; while defects in repair drive carcinogenesis, they also create exploitable weaknesses for targeted therapies [19,20]. For instance, PARP inhibitors exemplify the principle of synthetic lethality in BRCA-deficient cancers, leveraging tumor-specific DDR deficiencies to induce cell death [21,22]. Beyond PARP, emerging targets such as ATR, WEE1, and novel posttranslational modifications (e.g., UFMylation, crotonylation) are reshaping therapeutic strategies by modulating chromatin dynamics and repair protein interactions [23,24]. Meanwhile, single-cell genomics has revealed unprecedented heterogeneity in repair protein localization, linking spatial genome organization to repair outcomes [25,26].

The interplay between transcription and DNA damage repair further underscores the complexity of cellular responses. For example, bulky lesions like those induced by UV or cisplatin disrupt transcription elongation, yet active transcription is paradoxically required for damage signaling and repair [27]. Gene architecture—such as length, GC content, and exon density—has emerged as a critical determinant of transcriptional resilience, influencing how cells prioritize repair in transcribed regions [27,28,29]. Computational models now simulate these dynamics, predicting repair kinetics and mutational outcomes in diverse genomic contexts [30,31]. In parallel, the discovery of novel DDR biomarkers and pathways—such as replication stress in hematological malignancies or the role of APOBEC enzymes in generating mutation signatures—has expanded diagnostic and therapeutic horizons [32,33,34]. These advances not only deepen our mechanistic understanding but also pave the way for personalized interventions, from CRISPR-based editing safeguards to combinatorial regimens that amplify the efficacy of traditional chemotherapies [35,36,37]. This introduction synthesizes key breakthroughs in DNA damage research, emphasizing the integration of molecular insights, technological innovation, and clinical translation. As we unravel the intricacies of genome maintenance, the field stands poised to address longstanding challenges in precision medicine, aging, and disease prevention.

Cancer remains a leading cause of global morbidity and mortality, yet its burden is differentially distributed across populations, underscoring the pervasive challenge of health disparities. The drivers of cancer disparities are multifaceted, intertwining biological, environmental, and sociopolitical factors. Biological mechanisms, including genetic predispositions [38,39], e.g., BRCA mutations in certain ethnic groups [40], and tumor microenvironment variations, may interact with social stressors [41], such as chronic inflammation from systemic racism or environmental exposures in marginalized neighborhoods [42,43,44,45]. Meanwhile, structural barriers—discriminatory policies, insurance inequities, and implicit bias in clinical settings—compound these risks, limiting access to prevention, screening, and cutting-edge therapies [46,47,48]. Recent studies also highlight how epigenetic changes, driven by adverse social conditions, may amplify cancer susceptibility across generations [49,50,51].

Emerging research frameworks aim to dismantle these disparities through interdisciplinary approaches. Precision public health initiatives integrate genomic data with social context to tailor interventions [52], while community-based participatory research empowers affected populations to shape study design and outreach [47,48,53]. Advances in big data analytics now enable the mapping of disparities at granular levels, identifying “hotspots” of unmet need, and informing policy reforms [54,55]. Furthermore, investigations into the biological embedding of social adversity [56]—such as how chronic stress accelerates DNA damage or immune dysfunction [57,58,59]—are bridging the gap between molecular oncology and social epidemiology.

Therefore, different incidences, treatment access, and outcomes across populations are influenced by complex interactions between biological, environmental, and social factors. DNA damage studies provide critical insights into these disparities by unraveling how genetic instability, repair deficiencies, and environmental exposures disproportionately affect marginalized groups. Here, we discuss the key factors that eventually drive cancer susceptibility and lead to cancer health disparities, examining how they operate in the real world to create cancer health disparities, as seen among Native Hawaiians and Pacific Islanders (NH/PI).

## 2. Genetic and Epigenetic Drivers of Cancer Susceptibility

The susceptibility to cancer is driven by a dynamic interface of genetic alterations (both inherited and acquired) and modifiable epigenetic states. Elucidating these mechanisms advances risk stratification, early detection strategies, and the development of novel therapies directed at these combined vulnerabilities—thereby addressing the biological underpinnings of cancer health disparities.

### 2.1. Roles of Genetic Mutations (Inherited and Acquired) in Cancer Susceptibility

Genetic drivers are mutations or alterations in genes that directly contribute to the development of cancer. They drive the progression of cancer by giving cells a growth advantage [60,61]. Cancer susceptibility refers to how likely someone is to develop cancer based on their genetic makeup [62,63]. Cancer is a genetic disease caused by mutations. These mutations can be inherited or acquired. Inherited mutations are germline mutations passed down from parents, increasing cancer risk. Examples like BRCA1 and BRCA2 come to mind, which are linked to breast and ovarian cancers [64,65]. Acquired mutations are somatic, occurring in specific cells during a person’s life due to environmental factors like UV radiation or carcinogens [66]. These mutations accumulate over time, leading to cancer. Oncogenes and tumor suppressor genes are key here [67,68,69,70,71,72,73]. Oncogenes, when activated, promote cell growth. Examples include HER2 or RAS. Tumor suppressor genes, when inactivated, fail to regulate cell growth, like TP53 or APC. Also, chromosomal instability, copy number variations, and epigenetic changes like DNA methylation, like other genetic drivers [74,75,76,77,78,79,80,81], can all contribute to cancer susceptibility by disrupting normal gene function.

DNA damage studies reveal how inherited, acquired genetic variations or their interactions in DNA repair pathways (e.g., homologous recombination, mismatch repair) contribute to cancer health disparities [82,83,84,85,86,87]. These inherited (germline) and acquired (somatic) genetic variations distinctly influence critical phases of cancer biology, including susceptibility, tumor aggressiveness, and therapeutic response. As mentioned before, germline mutations, such as BRCA1/2 or TP53 variants, heighten baseline cancer risk by impairing DNA repair or tumor suppression. Somatic mutations, like oncogenic KRAS or EGFR alterations, drive tumor initiation and progression. During the aggressiveness phase, germline deficiencies (e.g., Lynch syndrome) amplify genomic instability [88], while acquired mutations in metastatic genes (e.g., MYC amplification) fuel invasive behavior [89]. Treatment outcomes are similarly shaped by both variation types: germline polymorphisms in drug-metabolizing enzymes (e.g., DPYD) modulate toxicity [90], whereas somatic alterations (e.g., BRCA reversion mutations) dictate therapeutic resistance [91]. This interplay underscores the dual genetic landscape shaping cancer disparities and precision oncology challenges. Germline variations were inherited from parents and present in reproductive cells (sperm/egg) and thus in every cell of the offspring; while somatic variations are acquired during an individual’s lifetime, occurring in non-reproductive cells (e.g., skin, liver) and affecting only descendant cells of the mutated cell. This basic distinction allows them to play a different role in the pathophysiology of human diseases, including cancer. Germline variations are linked to hereditary conditions and cancer predispositions, e.g., BRCA-related cancers [92]. Somatic or acquired variations then drive cancers and age-related diseases, often accompanying additional mutations to manifest. While both types of genetic variations involve DNA changes, germline mutations influence heredity and systemic traits, whereas somatic mutations drive localized diseases and are not inherited. Understanding both is crucial for personalized medicine and genetic counseling.

Germline mutations predispose individuals to cancer by elevating their baseline risk—the inherent likelihood of developing malignancies over a lifetime. Unlike somatic mutations, germline defects often disrupt critical pathways such as DNA repair (*BRCA1/2*), cell cycle control (*TP53*), or tumor suppression, creating a permissive genomic landscape for cancer initiation, contributing to cancer health disparities among the population carrying such inherited mutations.

### 2.2. Roles of Epigenetic Alterations in Cancer Susceptibility

Epigenetic alterations, the changes in gene expression without DNA sequence modifications, are pivotal in cancer susceptibility. Key mechanisms include DNA methylation, histone modifications, non-coding RNAs, and chromatin remodeling [93,94,95,96,97,98,99,100]. These processes, influencing oncogene activation, tumor suppressor silencing, and genomic instability, offer reversible targets for intervention. Advances in technology and combination therapies hold promise, though challenges in specificity and causality remain. Ongoing research underscores the dynamic interplay between environment, epigenetics, and genetics in cancer risk [29,100,101,102,103]. DNA methylation involves the addition of methyl groups to cytosine bases at CpG sites, typically repressing gene expression. This epigenetic modification regulates critical processes like cell differentiation and genomic stability. Aberrant methylation—such as hypermethylation of tumor suppressor genes or hypomethylation causing genomic instability—is a hallmark of cancer, contributing to uncontrolled cell growth and malignancy. Histone modifications are chemical changes (acetylation, methylation, phosphorylation, and ubiquitination) to histone proteins around which DNA is wrapped, altering chromatin accessibility. Non-coding RNA regulation is a biological process to silence genes or influence chromatin state by RNA molecules (e.g., microRNAs, lncRNAs). These epigenetic modification mechanisms can be initiated and/or enhanced by the exposure of numerous abnormal living environments, developmental and life stages, lifestyle factors, biological and physiological factors, stochastic (random) events, and even inheritance—transgenerational epigenetic inheritance (TEI) [104]. In the meantime, we need to be aware of some features associated with epigenetic modification, including the sensitive period (especially prenatal and early postnatal), potentially reversible epigenetic marks, exposure dose and duration, factor interplay, cell tissue specificity, etc. Understanding these factors is critical for grasping how environment and lifestyle interact with genetics to influence health, disease susceptibility, development, and aging. Based on the current literature, the updated understanding of factors influencing oncogenic epigenetic modifications highlights a multifaceted interplay of environmental, viral, lifestyle, and intrinsic biological factors. Environmental exposures, including ionizing radiation, induce aberrant DNA methylation (global hypomethylation and promoter-specific hypermethylation) and histone modifications, silencing tumor suppressors and activating oncogenes.

## 3. Social Factors or SDoH and Cancer Health Disparities

Social determinants of health (SDoH)—the conditions in which people are born, grow, live, work, and age—are fundamental drivers of persistent and inequitable differences in cancer outcomes across populations [105,106]. These disparities manifest at every stage of cancer care, as mentioned before, for their interplays with genetic factors, etc. [98,99], from prevention to survivorship, and are often more powerful predictors of health than genetic code or medical care alone [106,107]. A landmark 2022 study provides some of the most compelling evidence for the cumulative impact of SDoH. Analyzing data from over 29,000 participants, researchers found a direct “dose–response” relationship: as the number of adverse social determinants increases, so does the risk of dying from cancer [108]. Also, we would like to mention a retrospective cohort study that uses the CDC Social Vulnerability Index (CDC-SVI) to examine how social determinants of health (SDoH) affect care and survival in pediatric head-and-neck cancer (HNC). The analysis includes 37,043 patients (aged ≤ 19 years) diagnosed between 1975 and 2017 from the SEER database. The CDC-SVI aggregates 15 variables into four themes: socioeconomic status (SES), minority-language status (ML), household composition (HH), and housing-transportation (HT). Outcomes include surveillance duration, survival time, advanced staging, and receipt of surgery [109]. This study is a timely and important contribution to the literature on pediatric cancer disparities. By applying the CDC-SVI to a large, long-term cohort, it provides robust evidence that social vulnerability—especially socioeconomic and minority-language factors—is associated with substantial reductions in care receipt and survival for children with HNC. While limitations inherent to retrospective SEER data exist, the findings underscore the urgent need to integrate SDoH screening into pediatric oncology practice and to design equity-focused policies that address the root causes of these disparities. Addressing these disparities requires moving beyond merely documenting them to implementing multilevel interventions [110]. While awareness and action are growing, critical gaps remain. Standardized, validated SDoH screening tools, specifically for oncology settings to reliably identify patient needs, are a primary need. Research must also evolve from studying single factors to using multilevel frameworks (like the social-ecological model) that can unravel the complex interplay between individual, community, and societal determinants. Finally, more evidence is needed on which specific interventions are most effective at mitigating identified social risks and improving concrete cancer outcomes like stage at diagnosis, treatment completion, and survival.

## 4. Cancer Health Disparities in the Real World

Having outlined and discussed the causes of cancer disparities both genetically and societally, in the following section, we analyze how these factors uniquely manifest and drive gastrointestinal cancer disparities among Native Hawaiian and Pacific Islander (NH/PI) communities.

### 4.1. Health Disparity in Gastrointestinal Cancers in Hawaii

**Figure 1:**
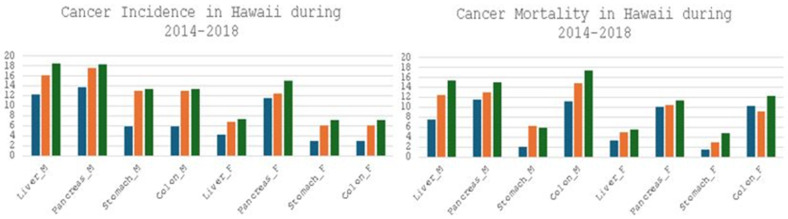
Male and female Native Hawaiians carry the highest rate for both incidence (**left**) and mortality (**right**) of colorectal, liver, pancreatic, and stomach cancers, compared to White and Japanese Americans. Blue bar—White, Orange bar—Japanese American, and Green bar—Native Hawaiian.

As the second most common cause of death in the US and many other countries around the world, cancer poses a significant health burden to our communities and major challenge to public health [111]. Substantial health disparities are present in cancer prevention and management, from risk reduction to disease detection, diagnosis to prognosis, and treatment to prevention. These disparities are also observed among Native Hawaiian and Other Pacific Islander (NH/PI) populations, who experience the highest cancer mortality (404.8/100,000) compared to the White (136.5/100,000) population, as documented by the Office of Minority Health. In Hawaii, cancer mortalities in both men and women are higher in the Native Hawaiian population than the White population. In addition, NH/PI show higher incidences and mortalities in four major gastrointestinal cancers (GICs) compared to other racial/ethnic groups [112,113]. NH/PI exhibited a higher incidence of colorectal cancer (CRC) in comparison to the general US population both in males (52.3 vs. 44.4/100,000) and females (37.1 vs. 34.0/100,000). NH/PI also had a higher mortality of CRC compared to the US population in males (20.2 vs. 16.6/100,000) and females (15.0 vs. 11.7/100,000) [114,115,116]. Other major GICs, i.e., liver, pancreas, and stomach, showed a trend similar to CRC. Furthermore, Native Hawaiians, both men and women, experienced a higher or the highest incidence and mortality in liver, pancreatic, colorectal, and stomach cancers compared to White and Japanese Americans in Hawaii (Hawaii Tumor Registry 2014–2018) (Figure 1). To address health disparities in major GICs in Hawaii, we need to identify factors that affect these cancers’ risk, diagnosis, treatment, and survival among NH/PI, and use the knowledge to develop and implement intervention strategies that can lower both the incidence and mortality of and improve health disparities in major GICs.

### 4.2. Factors Associated with the Risk of Major GICs

A combination of genetic, environmental, behavioral, and lifestyle factors have been identified to be associated with cancer risk [117]. These risk factors can be grouped into modifiable and non-modifiable categories [118,119,120]. The latter includes age, sex, family history of diseases, genetic variations and syndromes, and race/ethnicity. The former is often composed of dietary habits, physical activity, obesity, tobacco use, alcohol consumption, occupational hazards, socioeconomic factors, built environment, and health care policy and practice. Although each type of cancer has a unique risk profile involving different risk factors, some cancers share certain similarities in risk factors. For example, liver cancer is found to be associated with chronic viral hepatitis, heavy alcohol drinking, cigarette smoking, aflatoxin intake, obesity, and non-alcoholic fatty liver disease (NAFLD) or metabolic dysfunction-associated steatotic liver disease (MASLD) [121,122,123,124,125]. Pancreatic cancer risk is closely related to obesity, type 2 diabetes, cigarette smoking, chronic pancreatitis, and some genetic syndromes [126,127,128,129,130]. CRC risk factors include obesity, unhealthy diet, physical inactivity, smoking, drinking, family history, familial adenomatous polyposis (FAP), Lynch syndrome, and inflammatory bowel disease (IBD) [130,131,132,133,134]. Stomach cancer is associated with helicobacter pylori colonization, chronic gastritis, smoking, heavy drinking, obesity, unhealthy diet, and CDH1 mutation [130,135,136,137]. From the lists of risk factors, we can identify common ones, such as obesity, poor diet, physical inactivity, smoking, and drinking, in these GICs. Thus far, no study has addressed the common risk factors of GICs together in NH/PI, and moreover, there are limited investigations assessing the intersections between lifestyle risk factors and social determinants of health (SDoH), as well as their underlying mechanisms.

### 4.3. SDoH and Health Disparities in Major GICs

SDoH have been recognized to be important factors influencing disease risk, diagnosis, treatment, and outcome that lead to significant impacts on health equity [109,138]. Research has shown that people living under low socioeconomic conditions are more likely to adopt unhealthy lifestyles, to suffer from chronic diseases, to experience poor mental health, and to have limited access to health services and information, all of which collectively result in health disparities in cancer prevention, early detection, treatment choice, and survival outcomes [109,138,139,140]. With rising health burden worldwide in the major GICs, either due to aging population or increasing risk, identifying and understanding risk factors associated with or influenced by SDoH have become a pressing need in addressing cancer prevention, intervention, and survivorship [141,142]. Previous studies have indicated that certain SDoH, such as socioeconomic status, race/ethnicity, and transportation distance to a health provider, were important drivers of inequalities in disease screening, care delivery, and healthcare quality [138]. A large cohort study of childhood cancer survivors showed that patients with less education or low income were more likely to be obese and cigarette smokers, compared to those with more education or higher income [143]. Recently, large social and clinical data became available for researchers to assess the impact of SDoH on health disparities in a wide range of issues concerning health care [144,145,146].

### 4.4. Molecular Mechanisms Linking SDoH to Health Disparities in Cancer

SDoH are not only important in influencing health disparities in cancer diagnosis, treatment, and survival, but also intersecting with individual’s behaviors and lifestyles associated with cancer risk [46,109,147,148,149,150,151,152,153,154,155,156,157]. As social factors affect health across the entire life span [158], epigenetic research suggests that altered DNA methylation may underpin the link between health disparities and social adversities. Evidence shows that SDoH contribute to child mental health, asthma, and other health problems including health disparities [159]. Studies of biological underpinnings in SDoH have indicated epigenetics as a key molecular mechanism linking early life experience to health [158], connecting SES to inequities [160], and contributing to premature aging [161]. Although DNA sequences are the same for all the cells in each person, gene expression (mRNA) and functions in each cell are different and distinct. Epigenetic modification and regulation (epigenetics), e.g., DNA methylation, are important mechanisms underlying the differential gene expression and function [162,163,164]. Research into the relationships or interactions between social factors and epigenomics in health is called social genomics (or sociogenomics) [165]. DNA methylation and SDoH can influence each other in several ways. (a) Chronic Stresses: SDoH such as poverty, social inequality, or lack of access to healthcare can lead to chronic stress. Prolonged stress is known to alter DNA methylation patterns, increasing the risk for diseases, including cancer [166,167,168]. (b) Nutrition and Environmental Exposure: Poor nutrition or exposure to environmental hazards that are common in people with high SVI can affect DNA methylation. For example, deficiencies in folate and other micronutrients can alter DNA methylation, affecting gene expression and disease risk [8,169,170]. (c) Transgenerational Epigenetic Inheritance: Social determinants, such as socioeconomic status, discrimination, and access to healthcare, can affect not only adults but also their offspring. Epigenetic changes, including those in DNA methylation, can be passed on from one generation to another, contributing to the perpetuation of health disparities [171,172,173]. (d) Unequal Exposure to Risk Factors: People living in communities with a high SVI are more likely to experience different levels of environmental and/or psychosocial stressors that can lead to changes in epigenetics including altered DNA methylation [80,174,175,176,177,178], which further influence cancer risk, tumor progression, and disease outcomes. Based on these findings, DNA methylation has emerged as a crucial biological link between SDoH and health disparities involving cancer risk, detection, treatment, and prevention.

### 4.5. Altered DNA Methylations in GICs

The molecular basis for GICs, like others, is complex and heterogeneous, through a multistep progression driven by changes in cell functions and activities involving oncogene activation and tumor suppression gene inhibition [15,70,71,179,180,181,182,183,184,185,186,187,188,189]. DNA methylation plays a crucial role in the development and progression of the major GICs, including colorectal, gastric, liver, and pancreatic cancers. Tumor suppressors MLH1 and APC are frequently silenced due to promoter hypermethylation [190,191]. Along with this specific hypermethylation, GICs often exhibit global DNA hypomethylation, which leads to the activation of oncogenes. These aberrant DNA methylation patterns lead to genetic instability, a hallmark of cancer [192,193,194]. Some GICs, particularly CRC, are associated with a subtype known as CIMP (CpG Island Methylator Phenotype) [195] where multiple tumor suppressor genes are methylated. This phenotype is often associated with worse prognosis and can help classify cancer subtypes for specific treatment [196,197]. Beyond protein-coding genes, non-coding regions, such as long non-coding RNAs (lncRNAs), are also subject to abnormal methylation in GICs [198]. These changes can further disrupt gene expression regulation and contribute to cancer initiation and progression. CRC frequently exhibits hypermethylation in genes like MLH1, MGMT, and CDKN2A, leading to microsatellite instability and impaired DNA repair function [199,200,201,202].

In gastric cancer, hypermethylation of p16INK4a, RASSF1A, MLH1, and CHD1 is commonly observed [203,204,205,206]. Hepatocellular carcinoma (HCC) also shows widespread hypermethylation in genes like p16INK4a and RASSF1, which are linked to liver tumorigenesis [207,208]. Pancreatic ductal adenocarcinoma (PDAC) is characterized by hypermethylation of p16INK4a, BRCA1/2, and APC [209,210,211], indicating the aggressive nature of PDAC and, thus, poor prognosis. To date, abnormal methylation can also be detected in serum and stool samples, which may serve as biomarkers for cancer diagnosis or prognosis. For instance, methylation of SEPT9 or SFRP2 has been proposed as the according blood or stool-based biomarker for colorectal cancer screening [212,213,214,215], and the CIMP phenotype in CRC is also found in blood and stool samples [196]. Further, targeting methylation in GICs, such as using DNA methyltransferase inhibitors (e.g., 5-aza-2′-deoxycytidine) to reverse hypermethylation, is a potential therapeutic strategy [216,217,218]. Clearly, the ongoing research on aberrant DNA methylation in non-NH/PI populations offers the potential for improving GIC management, but challenges remain in translating the research findings into clinical practice. Similar research focusing on GICs in NH/PI is extremely limited, especially concerning changes in DNA methylation patterns in tumor suppressor genes and DNA repair genes in relation to SDoH and GIC risk factors.

There is limited information on how SDoH contribute to health disparity in the major GICs among NH/PI in Hawaii, as well as whether SVI intersects with high-risk lifestyles and behaviors among Hawaii residents and NH/PI populations. Future studies addressing these issues will offer new insights into the connection between SDoH and cancer risk in a special population. Data on the combined effect of SDoH and personal risk factors in GICs and on aberrant DNA methylation in DNA damage repair, inhibition of tumor suppressor genes, and activation of oncogenes is also limited. Collectively, these future studies will generate valuable information not only on the intersection of SDoH and risk lifestyles and behaviors in Hawaii, but also their joint impact on DNA methylation, which affects the biological functions of DNA damage repair, tumor suppressors, and oncogenes in relation to GICs, offering root causes for cancer health disparities.

## 5. Perspective

### Bridging the Molecular and the Social—DNA Damage as a Unifying Lens in Cancer Health Disparities

**Figure 2:**
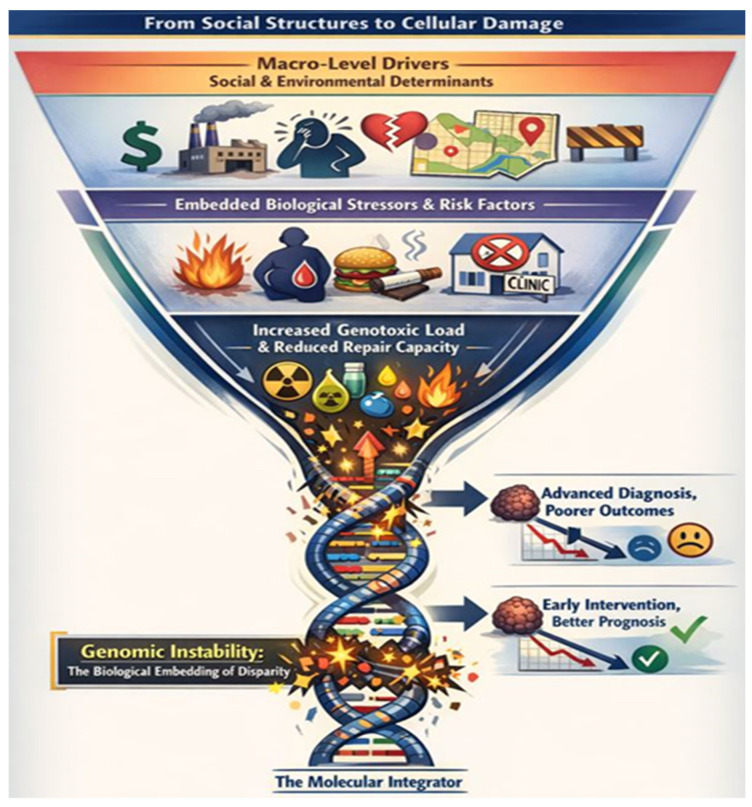
Tracing the Pathway from Society to the Cell: How Disparities Drive Genomic Instability. Disparate social and environmental exposures converge to create a higher burden of DNA damage in marginalized populations, which is directly expressed as the observed cancer health disparities.

Cancer health disparities—the profound inequalities in incidence, mortality, stage at diagnosis, and survivorship—are not merely statistical artifacts but manifestations of a complex hierarchy of causes (Figure 2). These range from macro-level social determinants (racism, poverty, and healthcare access) to individual behaviors and, ultimately, to molecular events within cells. A comprehensive understanding requires tracing the pathway from societal structures to biological consequence. Research into DNA damage and genomic instability provides a critical, mechanistic bridge across these levels. It allows us to unravel how the cumulative burden of social and environmental inequities becomes biologically embedded, leading to differential cancer susceptibility. Genetic variations in DNA repair pathways, while important, are only one piece of the puzzle. The more pervasive drivers of disparity often operate through differential exposure to genotoxic insults (e.g., environmental pollutants, dietary carcinogens, chronic inflammation from stress or infection) and differential capacity to mitigate damage (e.g., due to metabolic comorbidities, immune dysfunction, or access to preventative care).

The case of Native Hawaiians and Pacific Islanders (NH/PI) exemplifies this integration. Elevated rates of certain cancers in NH/PI populations cannot be attributed to genetics alone. Instead, they likely arise from the interplay of:

- Environmental Exposures: Historical and present-day environmental changes, including potential exposure to carcinogens from military activities or shifts in traditional land use.
- Social-Economic Factors: Systemic barriers leading to higher rates of obesity, diabetes, and tobacco use—all conditions that foster a pro-inflammatory state and genomic instability.
- Cultural and Systemic Barriers: Later-stage diagnosis due to disparities in screening access, healthcare distrust, or cultural misalignment of care, reducing the chances of intercepting premalignant damage.

Thus, DNA damage studies offer a powerful perspective: genomic instability is the molecular integrator of lived experience. It is the endpoint where historical trauma, socioeconomic disadvantage, environmental injustice, and biological vulnerability converge to increase cancer risk.

Moving forward, a disparities-focused research agenda must:

1. Embrace Translational Epidemiology: Link population-level exposure data with biomarkers of DNA damage (e.g., mutational signatures, micronuclei frequency) in disparity populations.
2. Contextualize Genetic Findings: Investigate how social and environmental factors modify the penetrance of common genetic variants in DNA repair genes.
3. Identify Intervention Points: Use the DNA damage framework to identify and prioritize modifiable risk factors—from policy-level environmental regulations to community-level screening programs.

By viewing cancer disparities through the lens of genomic instability, we can move beyond describing gaps to defining their precise etiologies. This perspective advocates for an interdisciplinary approach where molecular biology does not operate in isolation but is fundamentally informed by sociology, environmental science, and community-engaged research. Only through such integration can we develop equitable strategies for cancer prevention and precision oncology that truly address the roots of disparity.

## 6. Conclusions

Cancer health disparities are not inevitable but are the biological consequence of inequitable exposures and resources across populations. The framework of DNA damage and genomic instability provides a powerful unifying lens, tracing a direct pathway from macro-level social determinants—such as systemic racism, poverty, and environmental injustice—to the molecular chaos that defines malignancy. As exemplified by the elevated gastrointestinal cancer burden among Native Hawaiians and Pacific Islanders, disparities arise from the cumulative interplay of social adversity, behavioral risk factors, and biological susceptibility, which together accelerate DNA damage and impair protective cellular responses. Addressing these inequities therefore demands an integrated, interdisciplinary strategy that moves beyond isolated approaches. Future efforts must combine translational epidemiology, community-engaged research, and innovative biomarker studies to precisely identify modifiable risk factors along the social-to-biological continuum. By targeting the root causes of genomic instability, we can advance toward a more equitable paradigm in cancer prevention, early detection, and precision treatment, ensuring that scientific progress translates into just and measurable health benefits for all communities.
